# Supplementary material for: α-catenin switches between a slip and an asymmetric catch bond with F-actin to cooperatively regulate cell junction fluidity
Source: Nat Commun. 2022 Mar 3;13:1146. doi: 10.1038/s41467-022-28779-7 (PMC8894357; doi:10.1038/s41467-022-28779-7)
Supplement: Supplementary file 1 — Supplementary Information [file 41467_2022_28779_MOESM1_ESM.pdf]

α-catenin switches between a slip and an asymmetric catch bond with F-actin to cooperatively regulate cell junction fluidity

Arbore, C.*[,1,2], Sergides, M*[,1,2,3], Gardini, L.[1,4], Bianchi, G.[1,2], Kashchuk A.V.[1,2], Pertici I.[5], Bianco, P.[5], Pavone, F.S.[1,2,4], and M. Capitanio[#,1,2]

[1] LENS - European Laboratory for Non-linear Spectroscopy, University of Florence, Via Nello Carrara 1, 50019 Sesto Fiorentino, Italy.
[2] Department of Physics and Astronomy, University of Florence, Via Sansone 1, 50019 Sesto Fiorentino, Italy.
[3] Department of Physics, University of Cyprus, P.O. Box 20537, Nicosia, 1678, Cyprus.
[4] National Institute of Optics - National Research Council, Largo Fermi 6, 50125 Florence, Italy.
[5] Department of Biology, University of Florence, Via Madonna del Piano 6, 50019 Sesto Fiorentino, Italy.

*these authors contributed equally to this work
[#]Correspondence and requests for materials should be addressed to M.C. (email: capitanio@lens.unifi.it).

Supplementary Information

Supplementary figures

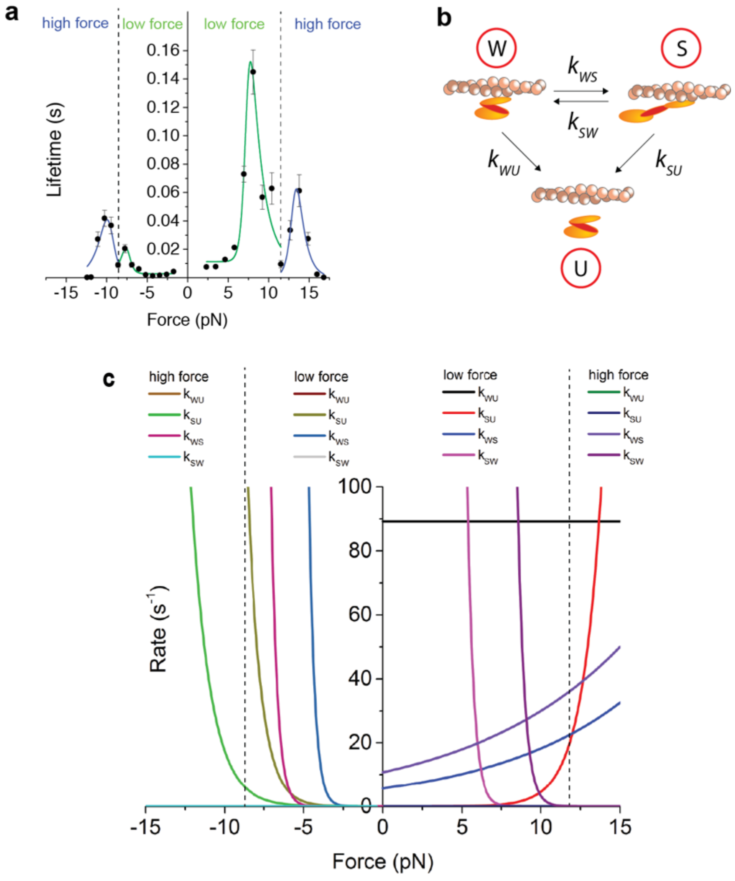

Supplementary figure 1: **Two-step catch-bond model**. **a**, The plot of the interaction lifetime between an α-catenin homodimer and actin vs force was divided into regions of low (green) and high (blue) force to separate the lifetime peaks. n = 23234 total number of interactions for all points in the plot. Error bars, s.e.m. **b**, Within each force region, α-catenin reversibly transitions between a weak, folded bound state (*W*) and a strong, unfolded state (*S*), with force-dependent rates k_{WS} and k_{SW}. Detachment from the bound states *W* and *S* to the unbound state *U* is described by the two rates k_{WU} and k_{SU}, respectively. Force-dependent rates have the form $k_{ij} = k_{ij}^{0}\exp\left(\frac{d_{ij}F}{k_B T}\right)$, where d_{ij} are the distance parameters and k^0_{ij} are the rates at no load. **c**, Plot showing the force-dependent rates obtained from fitting the two-state model to data in panel (a). Numerical values of the fit parameters are reported in Supplementary Table 1a.

a, single α-catenin homodimer

F > 0 low force

	Value (s^{-1})	Standard Error (s^{-1})		Value (nm)	Standard Error (nm)
k^0_{WU}	89	7	d_{WU}	0.00	0.04
k^0_{SU}	0.0014	0.00012	d_{SU}	3.31	0.04
k^0_{WS}	5.7	0.5	d_{WS}	0.47	0.04
k^0_{SW}	5.9E7	1.4E7	d_{SW}	-10.1	0.1

F > 0 high force

	Value (s^{-1})	Standard Error (s^{-1})		Value (nm)	Standard Error (nm)
k^0_{WU}	954	46	d_{WU}	1.61	0.01
k^0_{SU}	7.1E-8	0.5E-8	d_{SU}	3.15	0.02
k^0_{WS}	10.8	0.5	d_{WS}	0.42	0.01
k^0_{SW}	3.4E10	0.3E10	d_{SW}	-9.38	0.03

F < 0 low force

	Value (s^{-1})	Standard Error (s^{-1})		Value (nm)	Standard Error (nm)
k^0_{WU}	1000	1400	d_{WU}	0.0	0.7
k^0_{SU}	9.3E-4	4.5E-4	d_{SU}	-4.19	0.06
k^0_{WS}	1.5E-5	2.8E-5	d_{WS}	-11.0	0.2
k^0_{SW}	18000	30000	d_{SW}	0.0	0.2

F < 0 high force

	Value (s^{-1})	Standard Error (s^{-1})		Value (nm)	Standard Error (nm)
k^0_{WU}	80000	15000	d_{WU}	0.0	0.1
k^0_{SU}	0.00477	0.00036	d_{SU}	-3.35	0.03
k^0_{WS}	1.28E-6	0.25E-6	d_{WS}	-10.52	0.08
k^0_{SW}	1.4E8	3.9E8	d_{SW}	8.6	1.4

b,multiple α–β-catenin homodimers

F > 0 low force

	Value (s^{-1})	Standard Error (s^{-1})		Value (nm)	Standard Error (nm)
k^0_{WU}	110	20	d_{WU}	0.0	0.1
k^0_{SU}	0.061	0.008	d_{SU}	3.27	0.07
k^0_{WS}	0.52	0.15	d_{WS}	6.0	0.2
k^0_{SW}	1.3E6	0.4E6	d_{SW}	-5.0	0.2

Supplementary Table 1: **Two-step catch-bond model – fit parameters**. Force-dependent rates have the form $k_{ij} = k^0_{ij}\exp\left(\frac{d_{ij}F}{k_BT}\right)$, where d_{ij} are the distance parameters and k^0_{ij} are the

rates at zero load. **a**, Parameters of the force-dependent rates obtained from fitting the two-state model to the lifetime of a single α-catenin homodimer interaction (Fig. 1c). **b**, Parameters of the force-dependent rates obtained from fitting the two-state model to the lifetime of multiple single α–β-catenin heterodimers interaction (Supplementary figure 6).

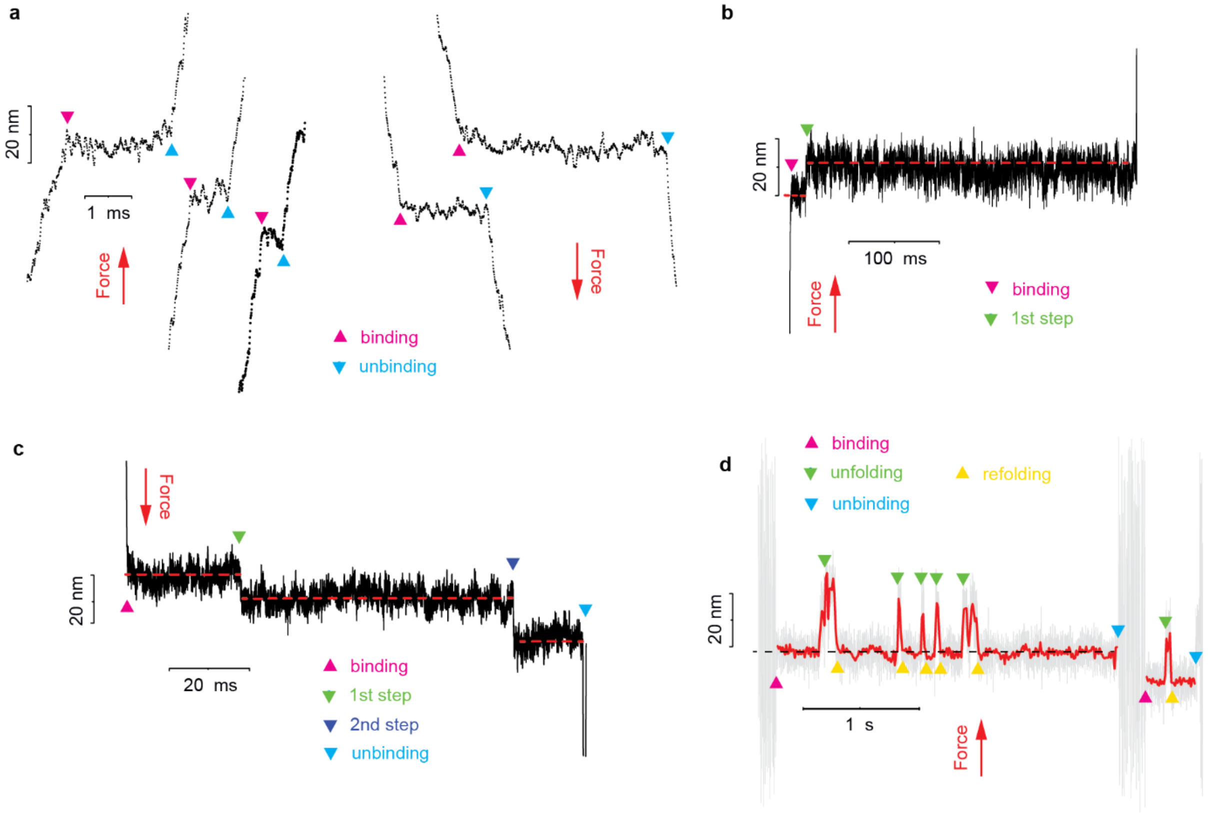

Supplementary figure 2: **Position records from α-catenin homodimers and α-β-catenin heterodimers under different forces**. **a,** An α-catenin homodimer under low force (~ 3 pN) shows single brief interactions; **b**, under moderate force (5 pN < *F* < 10 pN), α-catenin homodimer interactions show prevalently single steps; **c**, under high force (> 10 pN) α-catenin homodimer interactions usually display multiple steps. **d**, At about 5 pN force, a single α-catenin homodimer shows jumps between two positions.

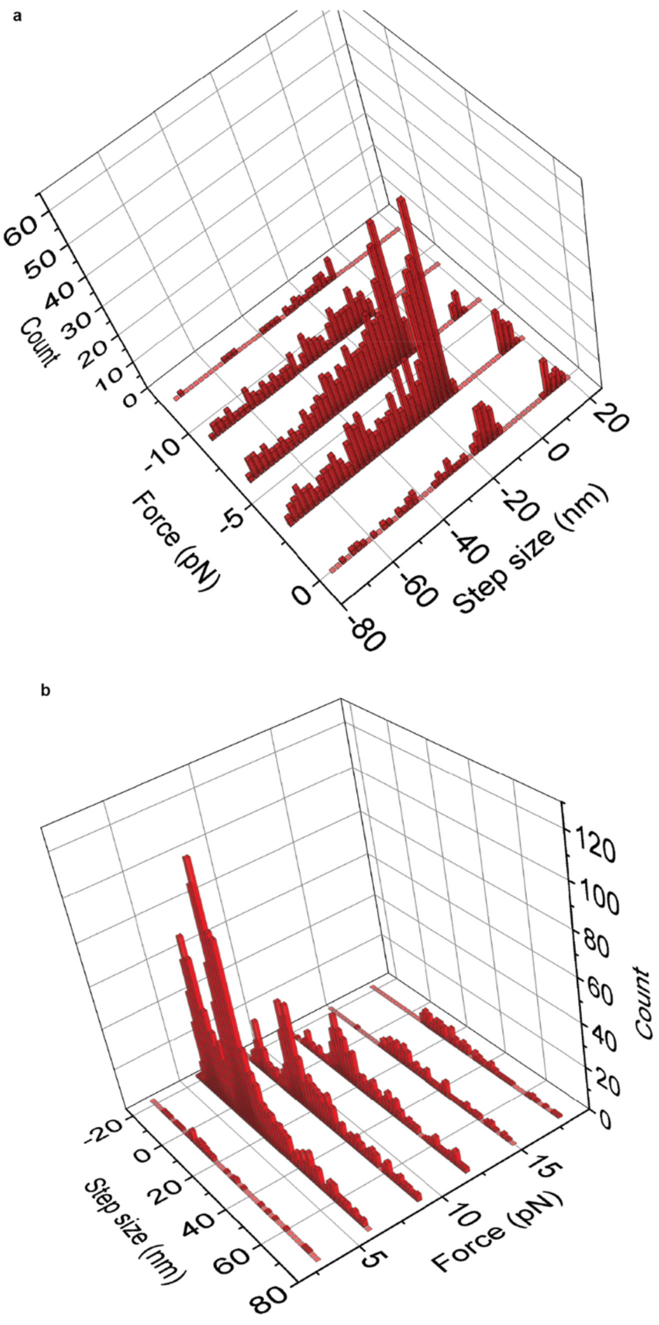

Supplementary figure 3: **Step size distribution versus force of a single α-catenin homodimer**. 3D plot of the step size distribution versus force for a single α-catenin homodimer molecule during its interaction with actin. **a**, Negative forces. **b**, Positive forces. The force sign is defined as in Figure 1. Source data are provided as a Source Data file.

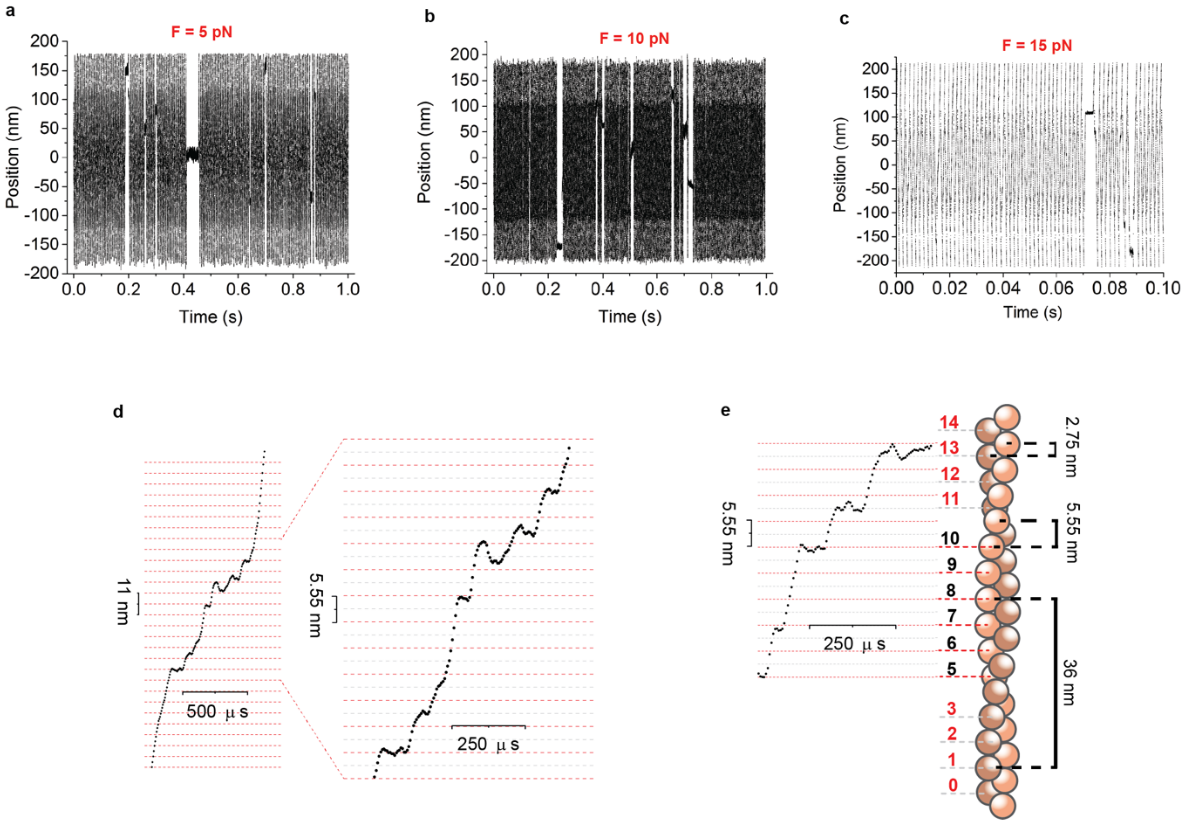

Supplementary figure 4: **Position records from single α-β-catenin heterodimers**. A single α-β-catenin heterodimer rapidly unbinds and rebinds from actin at all forces. Interaction lifetime decreases with force. **(a)** *F* = 5 pN, 1 s record; **(b)** *F* = 10 pN, 1 s record; **(c)** *F* = 15 pN, 0.1 s record. **(d)** Interactions are usually composed by a series of very rapid binding-unbinding sequences. As highlighted in Fig. 2d-f, binding occurs with a periodicity of 5.55 nm, which occasional shifts of 2.75 nm. **(e)** The figure shows that the binding periodicity of 5.55 nm is related to the distance between consecutive actin monomers, whereas the periodicity shift of 2.75 nm is a consequence of the distance between monomers on adjacent protofilaments. Traces in (d) and (e) are from experiments with *F* =15 pN.

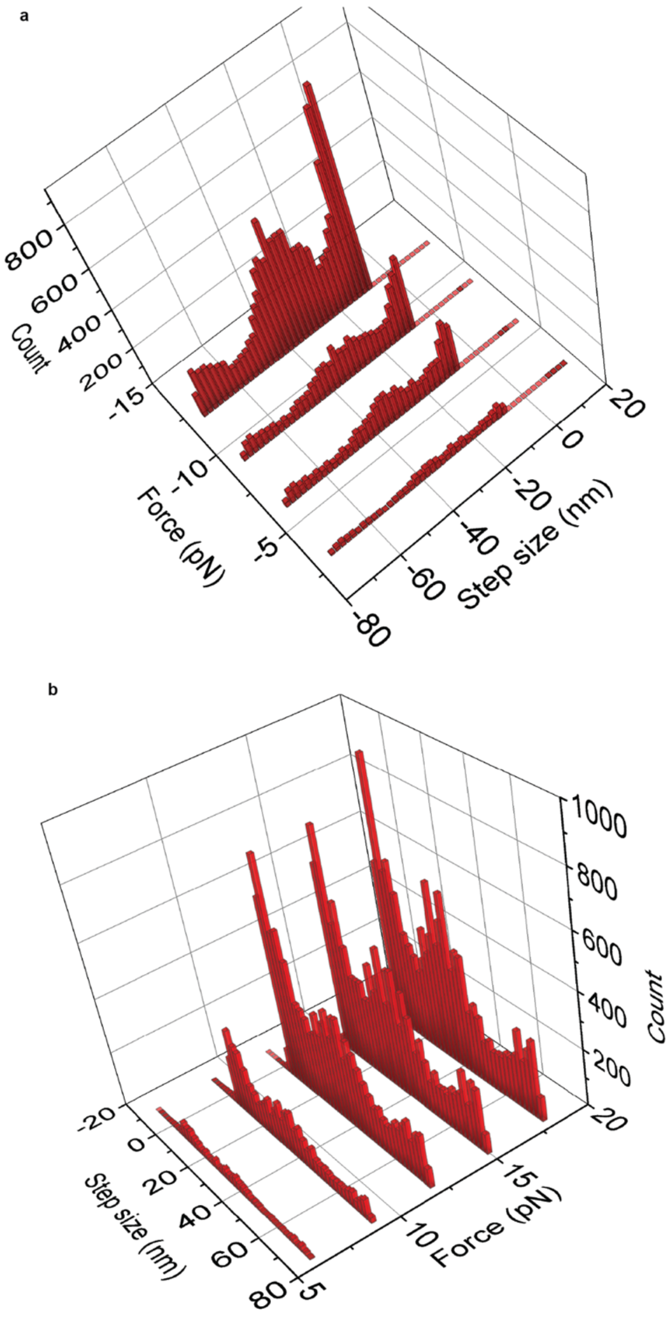

Supplementary figure 5: **Step size distribution versus force of a single α–β-catenin heterodimer**. 3D plot of the step size distribution versus force for a single α–β-catenin heterodimer during its interaction with actin. **a**, Negative forces. **b**, Positive forces. The force sign is defined as in Figure 2. Source data are provided as a Source Data file.

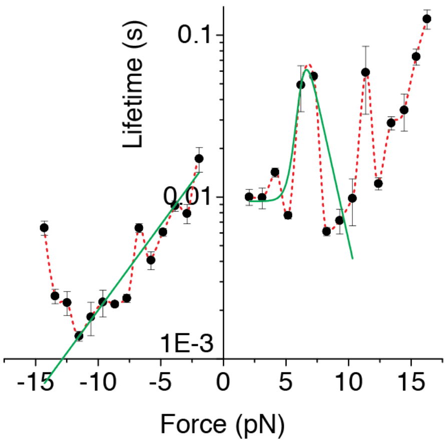

Supplementary figure 6: **Lifetime vs force for multiple α-β-catenin heterodimers**. Log-linear plot of the load-dependent lifetime of the interaction between multiple α-β-catenin heterodimers and actin. Green line is the fit of the peak occurring at lower force with the two-state catch-bond model at positive forces (see methods and Supplementary figure 1). Fit parameters are reported in the Supplementary table 1b. Fit of the peaks occurring at higher forces did not converge because of the low sampling of data. Blue line is the fit of lifetime τ with the Bell-bond equation $\tau = \tau_0 \exp\left(-\frac{d_{\alpha\beta}F}{k_B T}\right)$ (see Methods). Fitting parameters are $d_{\alpha\beta}$ = 1.0 ± 0.2 nm, τ_0 = 23 ± 5 ms. n = 55171 total number of interactions for all points in the plot. Error bars, s.e.m.

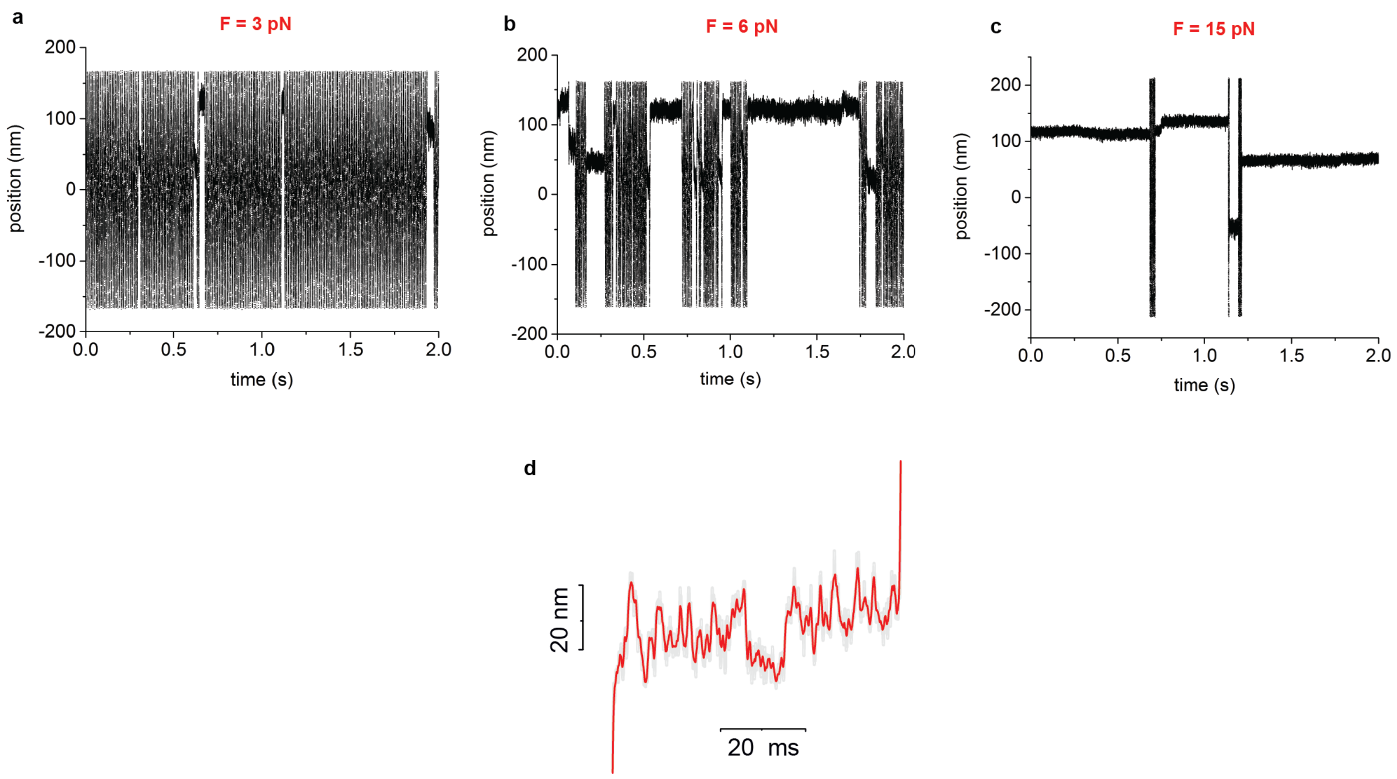

Supplementary figure 7: **Position records from multiple α-β-catenin heterodimers under different forces**. **a,b,c** Interactions of multiple α-β-catenin heterodimers with actin around 3 pN, 6 pN, and 15 pN, respectively. The lifetime of the interactions was significantly longer at 6 pN and 15 pN compared to 3 pN. **d**, Multiple α-β-catenin heterodimers at > 5 pN typically showed jumps between two positions.

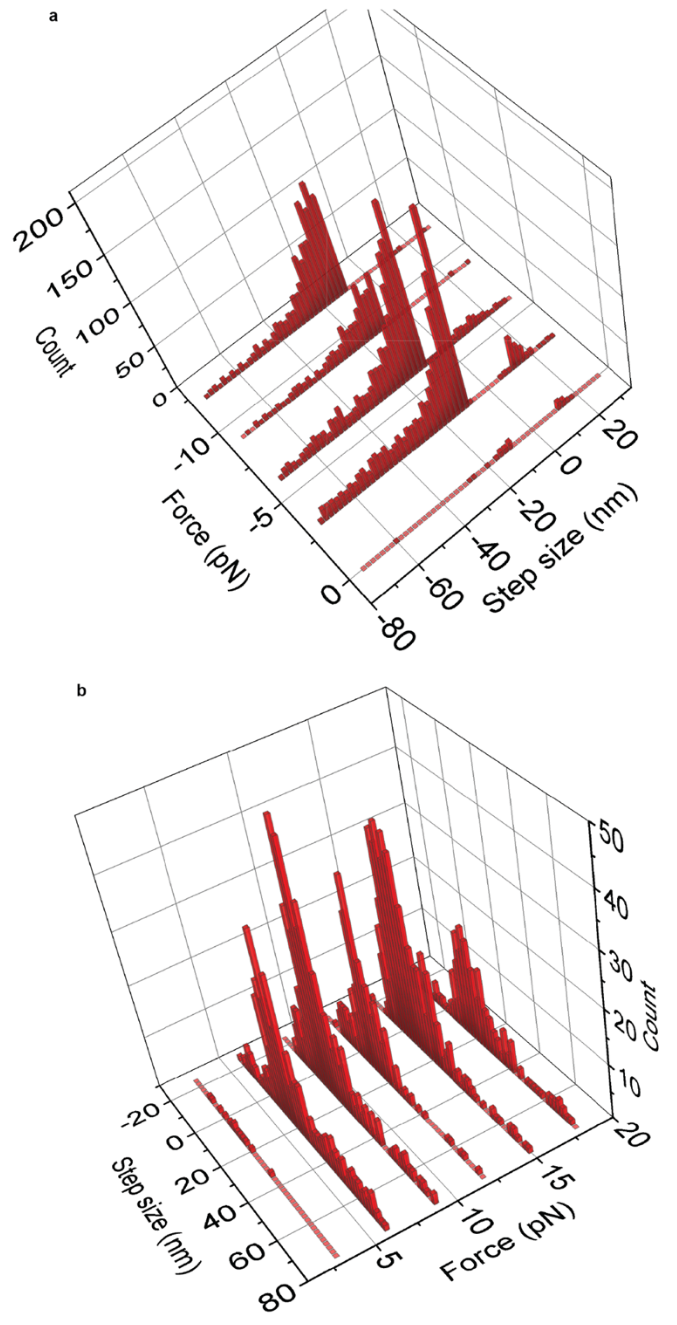

Supplementary figure 8: **Step size distribution versus force of multiple α–β-catenin heterodimer**s. 3D plot of the step size distribution versus force for multiple α–β-catenin complexes during their interaction with actin. **a**, Negative forces. **b**, Positive forces. The force sign is defined as in Figure 3. Source data are provided as a Source Data file.

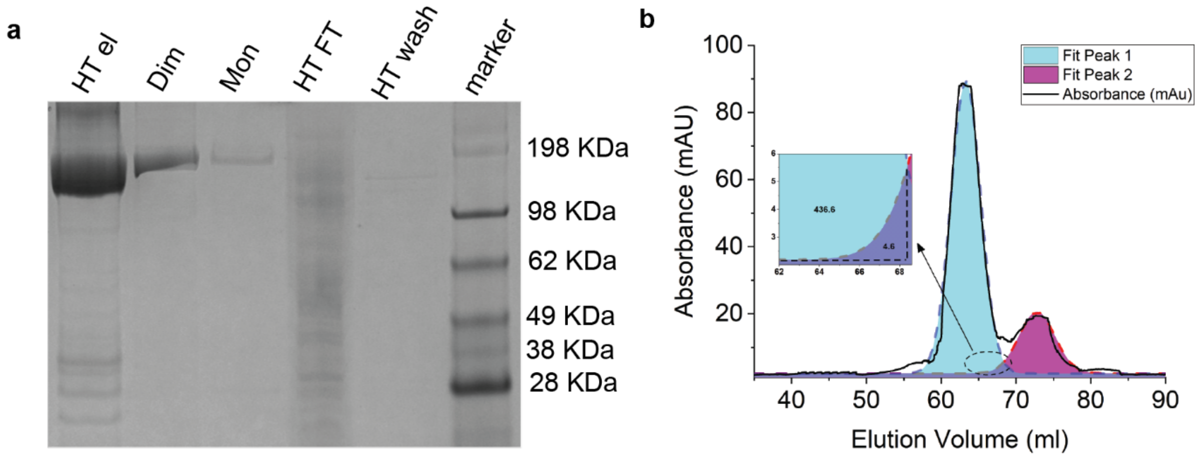

Supplementary figure 9: **α-catenin purification and dimer/monomer separation**. **a**, SDS-page gel, from the left: 1) elution of α-catenin from HisTrap columns; 2) dimeric and 3) monomeric fractions from size exclusion chromatography; 4) HisTrap flow-through; 5) HisTrap wash; 6) marker. Purification of α-catenin was repeated 4 times for the experiments reported here with similar results. **b**, Absorbance during elution from size exclusion column. The first peak from the left is from the dimeric α-catenin, the peak on the right from the monomeric α-catenin. From the area under the Gaussian fit of the two peaks (cyan and purple for the dimeric and monomeric catenin, respectively), we calculated the percentage of monomeric and dimeric catenin contained in each 1 ml elution aliquot. We pooled aliquots containing dimeric catenin with <1% monomeric catenin, and, similarly, monomeric catenin with <1% dimeric catenin. The figure inset shows the point where the monomeric catenin (area 4.6 AU) is about 1% of the dimeric catenin (area 436.6 AU).

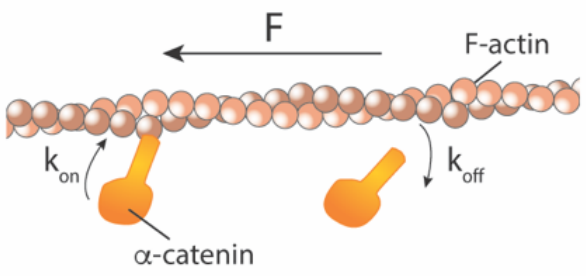

Supplementary figure 10: **α-catenin mechano-kinetic model.** Figure shows a sketch of the model of interaction of α-catenin with actin. The sliding velocity of the actin filament depends on the force applied to the filament (F) by active enzymes such as non-muscle myosin II and the force applied by α-catenin, which binds and unbinds from the filament with attachment and detachment rates k_{on} and k_{off}, respectively

Supplementary Methods

α-catenin-actin pull-down assay

A pull-down assay was used to confirm the predicted interaction between the His(6x) tagged α-E-catenin and F-actin and measure the affinity of the complex *in vitro.* With such a method, proteins are mixed in solution and binding is evaluated in the absence of any external applied force (F=0). Each reaction contained increasing concentrations of α-catenin incubated with a fixed concentration of F-actin. Following binding and pelleting of the α-E-catenin-actin complexes through centrifugation, the proteins recovered from individual pull-downs were

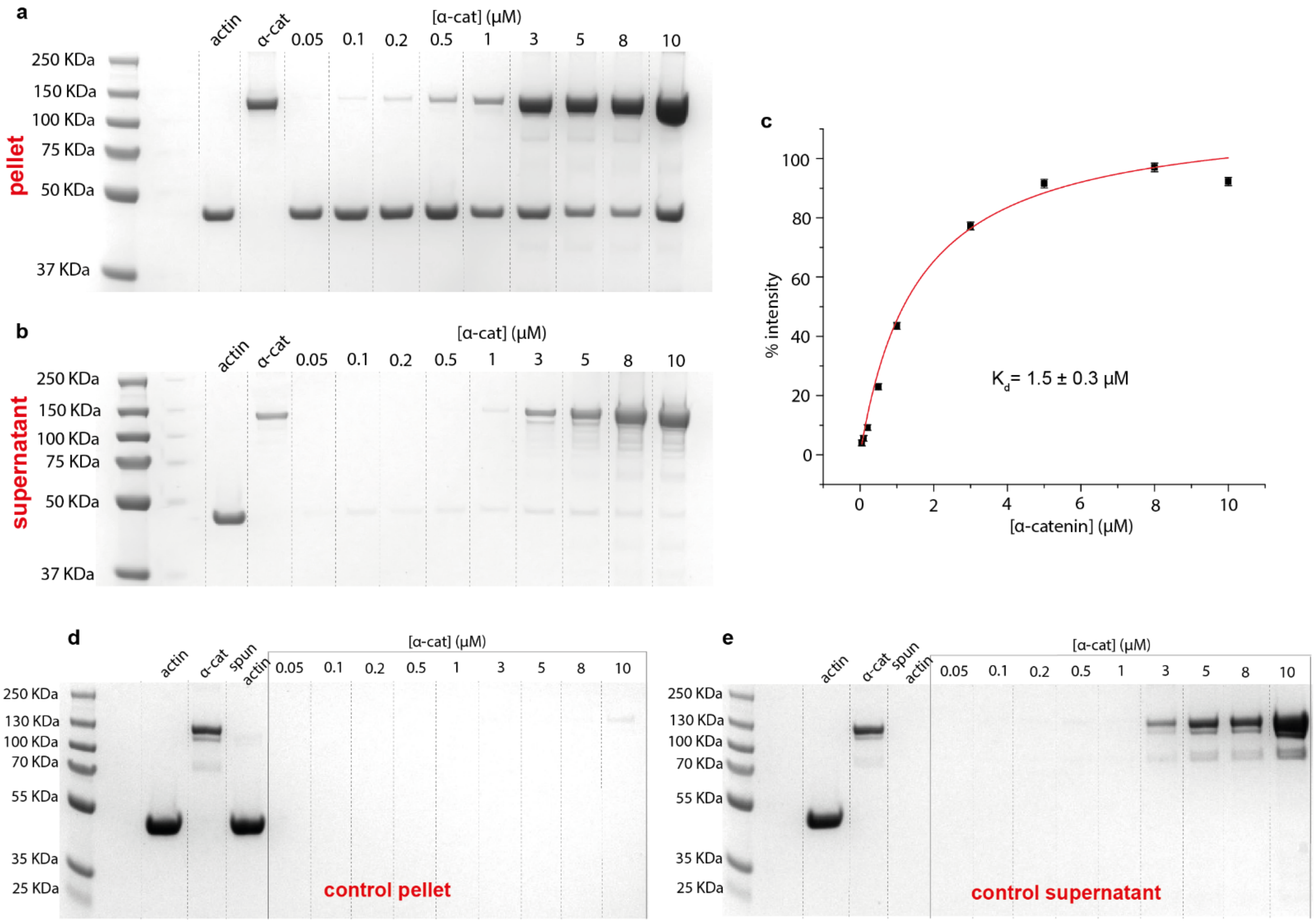

Supplementary Fig. 11: α-catenin-actin pull-down assay. a, SDS-page gel of the pellet of pull-down assay at increasing concentration of His(6x) tagged α-catenin. Lanes: 1, marker; 2, empty; 3-4, purified actin and α-catenin for reference; 5-13, pellet at increasing α-catenin concentration. **b,** SDS-page gel of the supernatant of pull-down assay at increasing concentration of His(6x) tagged α-catenin. Lanes: 1, marker; 2, empty; 3-4: purified actin and α-catenin for reference; 5-13, supernatant at increasing α-catenin concentration. Experiment was repeated two times with similar results. **c**, Densitometric analysis of the SDS-PAGE gel bands in (a) (black circles) was fitted with a Michaelis-Menten equation (red line). N=9 average intensity of the α-catenin band normalized to the average intensity of the actin band. Error bars are s.e. assuming Poisson distributed intensity. Source data are provided as a Source Data file. **d,e**, Respectively control pellet and supernatant of α-catenin and actin spun individually. Lanes: 1, marker; 2, empty; 3-4, purified actin and α-catenin for reference; 5, actin alone pellet (d) and supernatant (e); 6-14, α-catenin pellet (d) and supernatant (e)at increasing α-catenin concentration. Experiment was repeated two times with similar results.

analyzed on an SDS-PAGE by densitometry (Supplementary Fig. 11a,c). Reactions and conditions are described in Methods. The intensities of the bands corresponding to the fraction of α-catenin bound to F-actin were quantified for each concentration and normalized to the intensity of the corresponding actin band to correct for the variability in the amount of pellet collection[1]. The SDS-PAGE gel of the corresponding supernatant is shown in Fig. 11b as control. Control experiments in which α-catenin and actin were spun by themselves show that the fraction of α-catenin that pellet independently of actin is negligible (Supplementary Fig. 11d,e; in particular, compare lanes of α-catenin at 10 μM between panels a and d). Data were fitted by a Michaelis-Menten equation $I = \frac{I_{max} \cdot [\alpha cat]}{K_d \cdot [\alpha cat]}$, giving a dissociation constant K_d = 1.5 ± 0.3 μM (Supplementary Fig. 11c) , in good agreement with previous reports[2,3].

α-catenin-β-catenin pull-down assay

A pull-down assay was also used to confirm the predicted interaction between the His(6x) tagged α-E-catenin and the GST tagged β-catenin and measure the affinity of the complex *in vitro*[4]. GST-tagged β-catenin was covalently bound to carboxylated polystyrene beads at the protein N-terminus. Each reaction contained increasing concentrations of α-catenin incubated with a fixed concentration of 21.7 nM β-catenin. Following binding and pelleting of the α-E-catenin-β-catenin complexes through centrifugation, the proteins recovered from individual pull-downs were analyzed on an SDS-PAGE by densitometry (Supplementary Fig. 12a,c). Reactions and conditions are described in Methods. The intensities of the bands corresponding to the fraction of α-catenin bound to β-catenin were quantified for each concentration. The SDS-PAGE gel of the corresponding supernatant is shown in Fig. 12a,d for reference. Note that α-catenin concentration in the 400 μl of supernatant is less than 1/10 of α-catenin concentration in the 30 μl in which is resuspended the pellet (see Methods), which is probably why residual α-catenin is not visible in the supernatant of β-catenin beads. Pellet intensity data were well fitted by a Michaelis-Menten equation, giving a dissociation constant K_d = 35 ± 13 nM. This is in good agreement with previous measurements of K_d between untagged αE-catenin and untagged β-catenin by Pokutta et al. (23.4 ± 3.7 nM)[5], indicating that the His(6x) and GST tags are not affecting significantly the interaction between α-catenin and β-catenin. BSA control beads were used to test the influence of non-specific interactions between α-catenin and beads as in Lapetina et al.[4]. Analysis of pellet in control BSA beads shows that there is no correlation between the quantity of α-catenin that is pulled down together with β-catenin beads and α-catenin that is pulled down together with control beads. Therefore, non-specific interactions between α-catenin and beads and the fraction of α-catenin that is pulled down in the absence of β-catenin can be neglected[4].

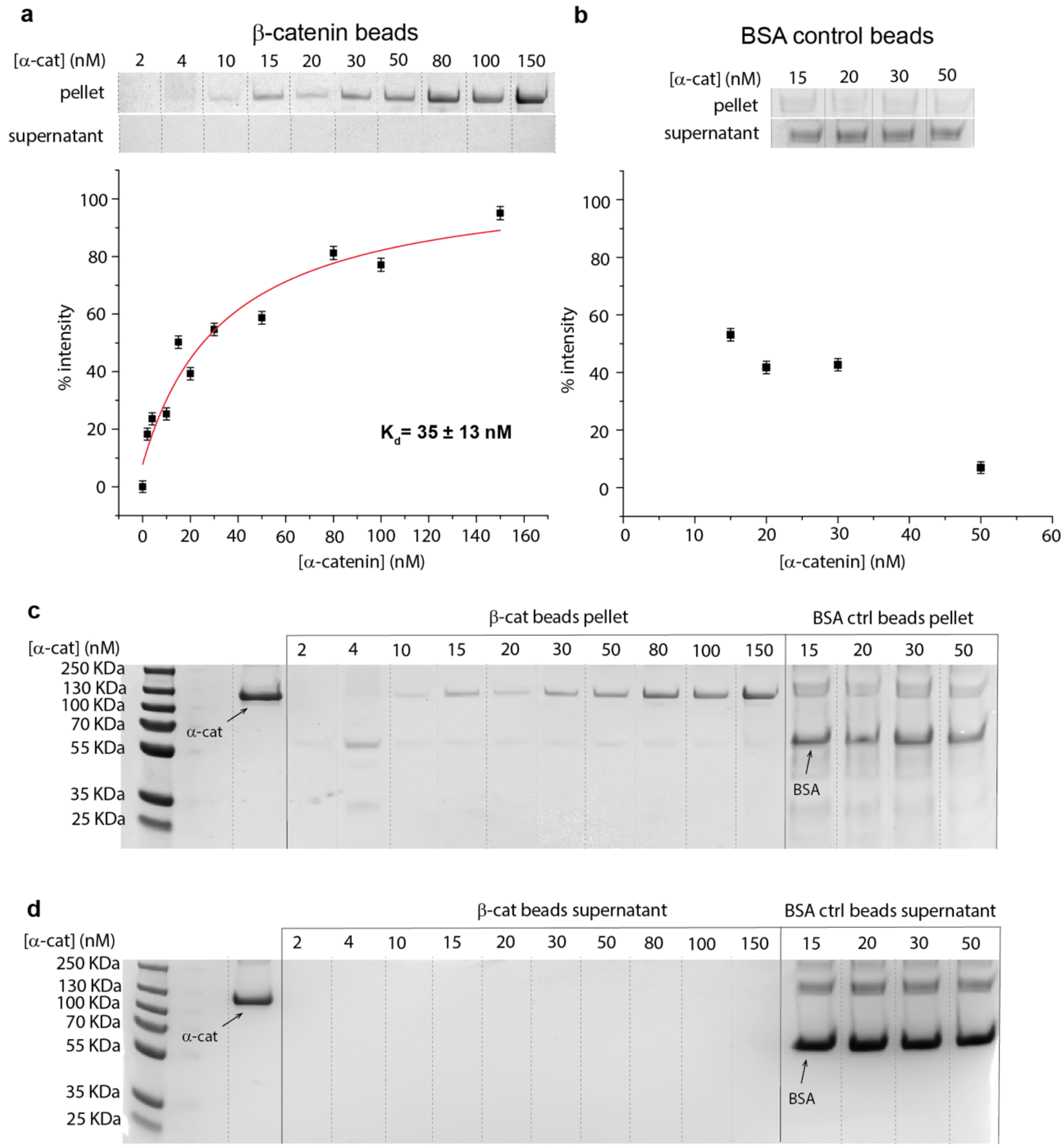

Supplementary Fig. 12: α-catenin-β-catenin pull-down assay. a, Top, SDS-page gel of α-catenin in the pellet and supernatant from pull-down assay between GST-β-catenin and His(6x)-α-catenin at increasing concentrations (full-length gel image in panel c and d). Bottom, Densitometric analysis of the SDS-PAGE gel bands of pulled-down α-catenin (black circles) was fitted with a Michaelis-Menten equation (red line). N=11 average intensity of the β-catenin band. Error bars are s.e. assuming Poisson distributed intensity. Source data are provided as a Source Data file. **b,** Top, SDS-page gel of α-catenin in the pellet and supernatant from pull-down assay of BSA control beads at increasing α-catenin concentration (full-length gel image in panel c and d). Bottom, Densitometric analysis of the SDS-PAGE gel bands of pulled-down α-catenin in control experiments. N=4 average intensity of the β-catenin band. Error bars are s.e. assuming Poisson distributed intensity. **c**, Full-length gel of pellets. **d**, Full-length gel of supernatants.

Flow cell assay

Binding activity of α-catenin to F-actin was then evaluated through a flow cell assay to test whether α-catenin homodimers and α–β-catenin heterodimers could bind to actin under force. As in all experiments, we used His(6x) tagged α-E-catenin and GST tagged β-catenin. In this assay, α-catenin was first attached to the coverslip surface either on top of nitrocellulose or over a β-catenin bed. Next, fluorescently labelled F-actin was flowed and incubated into the chamber and then washed with an imaging buffer to remove floating F-actin and observe whether F-actin on the coverslip surface remained bound to α-catenin under the drag force applied by the buffer flow. The force applied to an actin filament by the buffer flow can be calculated as[6]:

$$\frac{F}{l} = c_{\parallel} v = \frac{2\pi\eta}{ln(2h/r)} v$$

where l is the length of the actin filament, $c_{\parallel}$ is the drag coefficient per unit length along the actin filament axis, v is the flow velocity (~2 mm/s), η is the coefficient of viscosity of the buffer (~10^{-3} N·s/m), h is the distance between the coverslip surface and the center of the actin

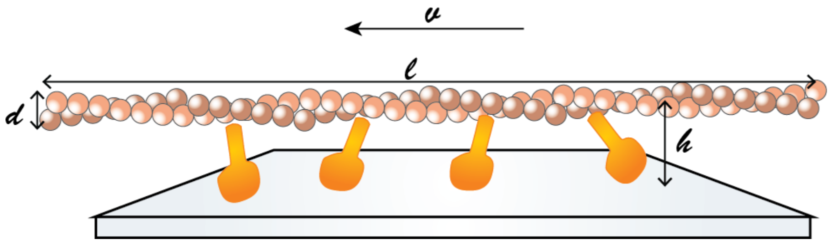

Supplementary Fig. 13: α-catenin flow cell assay. Figure shows a sketch of the experimental configuration of the flow cell assay indicating the distance between the coverslip surface and the center of the actin filament (h), the filament diameter (d), the length of the actin filament (l), and the buffer flow velocity.

filament (~10 nm), and r is the filament radius (~3 nm)[6] (Supplementary Fig. 13). From this formula we estimate a force per unit length of about 7 pN/μm. Given filament lengths in the range 1 - 10 μm, the flow cell assay allowed us to observe if multiple α-catenin molecules could bear forces of few tens of piconewton on actin.

Supplementary Fig. 14a and 14b show a field of view of a flow cell experiment in which α-catenin homodimers were bound onto a nitrocellulose smeared coverslip at concentration of 1 μM and 0.4 μM, respectively. Actin filaments bound on the surface were visible after the washing step in both conditions, with a significantly higher number of filaments for the higher α-catenin concentration. Supplementary Fig. 14c show a field of view of a flow cell experiment in which α-catenin at 1 μM concentration was bound onto a bed of β-catenin, attached to a nitrocellulose smeared coverslip. Also under these conditions, actin filaments bound on the surface were visible after the washing step. A control reaction in the absence of α-catenin was also performed showing no detectable F-actin filaments on the surface (Supplementary Fig. 14d). Reactions and conditions are described in detail in Methods.

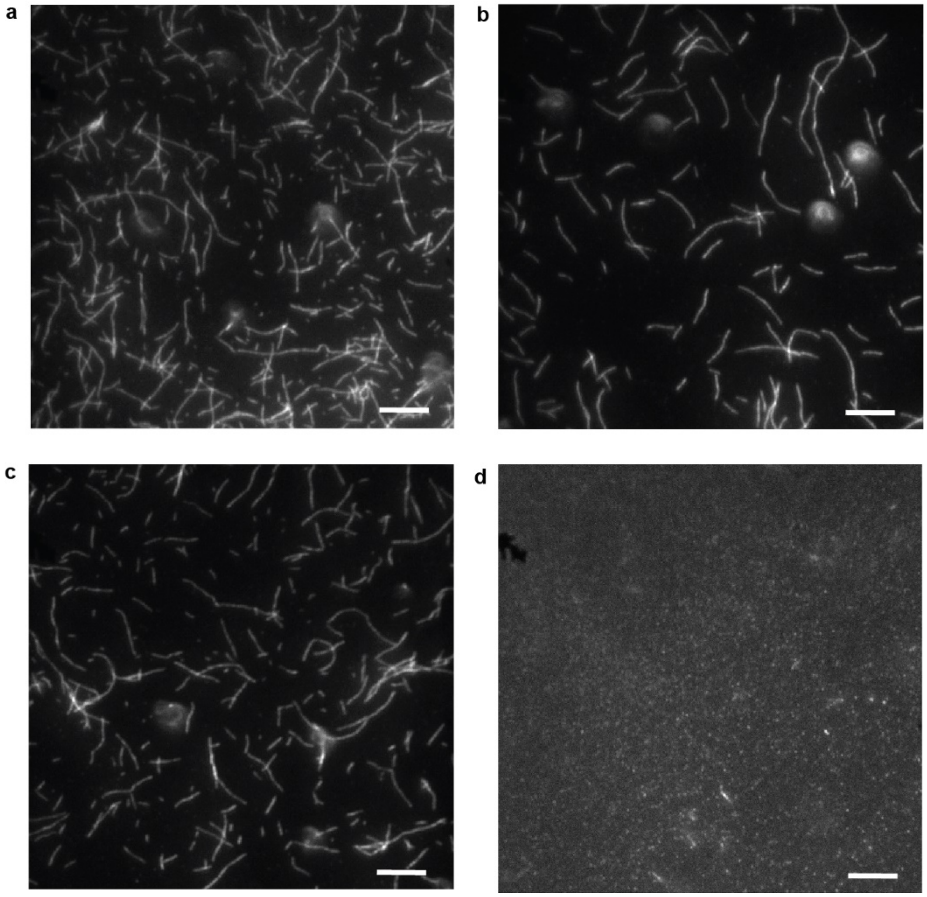

Supplementary Fig. 14: α-catenin homodimer flow cell assay. a, field of view of a flow cell experiment in which α-catenin homodimers at 1 μM concentration was bound onto a nitrocellulose smeared coverslip. Actin filaments bound on the surface were visible after the washing step. **b,** field of view of a flow cell experiment as in (a) but with α-catenin at 0.4 μM concentration **c,** field of view of a flow cell experiment in which α-catenin at 1 μM concentration was bound onto a bed of β-catenin, attached to a nitrocellulose smeared coverslip. **d**, Control reaction with 1 μM GST-β-catenin attached to the nitrocellulose smeared coverslip in the absence of α-catenin showing no detectable F-actin filaments on the surface. Scale bar is 5 μm. Experiments were repeated 3 times with similar results.

Supplementary discussion

Step Size Distribution

In our experiments, a single α-catenin homodimer might bind to the actin filament with a single monomer or with both. Binding of both monomers to actin would lead to the possibility that the position steps observed at force > 5pN are the consequence of the unbinding of one of the two monomers. In fact, monomer unbinding would result in the displacement of the actin filament under force as a consequence of the change in the bond stiffness. Similarly, a step might result from the unbinding of one monomer in the experiments with multiple α-β-catenin heterodimers. However, we believe that such events cannot be the primary cause of the main peak observed in the step distributions, for the following reasoning.
Assume that the stiffness of a single monomer bound to F-actin is k_m and the stiffness of the dimer bound to F-actin is $k_d = 2k_m$. Under a constant force F, the dimer would be strained by $x_d = F/k_d = F/2k_m$, whereas the monomer by $x_m = F/k_m$. Therefore, the step that we should observe when a monomer detaches from actin and the other stays bound is $d = x_m - x_d = F/2k_m$. Under this hypothesis, the d ~ 12 nm step measured at F ~ 5 pN force would imply that the stiffness of the monomer is about k_m = 5 pN/(2*12 nm) = 0.21 pN/nm. At 11 pN force, the unbinding of one monomer would produce a step $d = F/2k_m$ = 11 pN / (0.42 pN/nm) = 26 nm, whereas we observe a main peak in the step distribution of similar size.
Therefore, in our opinion the most plausible explanation is that the step is due to a conformational change of the protein (unfolding) that does not modify substantially the protein stiffness. Under this hypothesis, since the applied force is constant and the protein is already strained by the force before the unfolding occurs (force application is much faster than the step dynamics[7]), the unfolding step would be independent of the applied force, as we observe.
It still remains the possibility that unbinding of one of the two monomers contributes to the larger steps that we see at larger forces. For example, at 11 pN force we observe a second peak around 35 nm, which might fit the sum of the unfolding step (12 nm) and monomer unbinding (26 nm).

Cooperative binding

It's well established that that the interaction between α-β-catenin heterodimers and actin is much weaker than between α-catenin homodimers and actin[8]. Different explanations have been proposed previously (i) the N- and C-terminal domains of α-catenin are allosterically coupled and binding to β-catenin on the N-terminal domain might alter the C-terminal domain ability to bind to actin[8]. (ii) Structural studies[9] indicate that β-catenin might sterically hinder F-actin binding by the α-catenin binding domain, which could be at the basis of the different F-actin binding between the homodimer and heterodimer. (iii) α-catenin ABD binding to actin is accompanied by a conformational change in the actin protomer that affects the filament structure. This alteration of the filament structure can be at the base of a cooperative binding mechanism that reinforces the link between an α-catenin homodimer and actin compared to an α-catenin monomer[3]. Our results indicate that a cooperative mechanism is at the basis of the bond reinforcement and the analysis of the α-catenin stiffness during the interaction with actin in the different experiments reinforces this interpretation (see discussion in the main text).

However, the identification of the structural features that are at the basis of the different kinetics of α-catenin homodimers and heterodimers is out of the scope of our article, and further studies would be required to clearly assess this point.

Non-specific interactions

We made many control experiments to rule out non-specific interactions, which are one of the well-known issues in this kind of single molecule experiments. In experiments on α-β-catenin heterodimers, α-catenin was attached on the coverslip surface on top of a nitrocellulose-coated surface saturated with GST-β-catenin, followed by BSA (see methods). We made several control slides in which the coverslip surface was coated as described above but in the absence of α-catenin and looked for non-specific interactions on several tens of beads in each slide. We could very rarely (less than one bead per slide) find non-specific interactions with this control surface. Moreover, non-specific interactions were very different from the interactions observed in the presence of α-catenin, showing few short interactions when the dumbbell was close to the coverslip surface and the actin filament was pushing on the bead (as detected from the change in the position signal) and disappeared when the dumbbell was moved slightly farther from the coverslip surface. On the other hand, in the presence of α-catenin at single molecule concentration, we observed interactions in one every 4 beads on average, the interactions were much longer at low forces (tens of milliseconds) and the number of interactions increased with force. A single molecule was able to produce as much as several tens of thousands interactions. The interactions were observed also when the actin filament was not pushing on the bead. This behavior was never observed in the absence of α-catenin.

Dimeric vs monomeric α-catenin

Before our experiments, we separated dimeric from monomeric α-catenin by using size exclusion chromatography. This procedure assures that less than 1% of the catenin was in a dimeric form in the experiments with α-β-catenin heterodimers (see supplementary Fig. 9). The concentration of the monomeric catenin that we used in the experiments at single molecule concentration was about 1 μg/ml (10 nM); in the ones at “high” concentration, catenin concentration was 10 μg/ml (100 nM). Since the dissociation constant of the α-catenin homodimer is 25 μM, at equilibrium about 0.04% and 0.4% would be dimeric at the single-molecule and high concentrations, respectively. Moreover, Pokutta et al. showed that the α-catenin homodimer does not bind to β-catenin even after overnight incubation[5]. Therefore, the few % contamination of dimeric catenin was most likely washed away after few minutes of incubation in the sample chamber (see methods).

Possible effect of the GST tag in β-catenin

In the experiments reported in this work, we used GST-tagged β-catenin. The presence of the GST tag might in principle influence the interaction of β-catenin with α-catenin and actin. Although previous studies indicate that the GST tag does not introduce non-specific interactions with actin[2], we directly tested whether the GST tag might introduce non-specific interactions with actin, α-catenin, or both.

We checked non-specific interactions between the GST-tagged β-catenin and actin using a flow cell assay and single molecule experiments. Using the flow cell assay, we did not observe binding of actin filaments on the coverslip surface coated with GST-tagged β-catenin (see Supplementary Methods, section “flow cell assay” and Supplementary Fig. 14d). In single

molecule experiments, we made several control coverslips coated with the GST-tagged β-catenin and very rarely observed non-specific interactions with an actin filament, similarly to what is observed in the absence of the GST-tagged beta-catenin (see methods, section “Optical trapping experiments” and supplementary discussion, section “non-specific interactions”).

We also performed experiments to evaluate whether the presence of the GST tag would affect the interaction between β-catenin and α-catenin. To this end, we made pull-down experiments in which we bound GST-β-catenin to microbeads and made them react with α-catenin at growing concentrations. From these experiments, we measured a dissociation constant between α-catenin and β-catenin of K_d = 35 ± 13 nM (see methods, section “Pull down and flow-cell assay”, and supplementary materials, section “α-catenin-β-catenin pull-down assay”). Previous work by Koslov et al. (1997) determined k_d of 100nM between untagged full-length alpha-E-catenin and untagged full-length beta-catenin, but no experimental error was reported on this value[10]. Later, Pokutta et al. (2014) determined k_d = 23.4 ± 3.7 on the same constructs, which is in good agreement with our measurement[5]. This result indicates that the interaction between α-catenin and β-catenin is not affected significantly by the presence of the GST tag.

Another aspect that should be taken into account is that the GST tag can form dimers, which might in principle influence the single molecule or cooperative action of α-β-catenin heterodimers. However, in our experiments we used β-catenin to coat the coverslip surface at saturating concentration and we bound α-catenin on top of this β-catenin carpet at lower concentration. Under this condition, we expect to find β-catenin molecules tightly packed on the coverslip surface regardless of their dimeric or monomeric state and α-catenin distribution on the surface dictated mostly by α-catenin concentration, not by β-catenin dimeric or monomeric state.

In support to this argument, we have strong experimental evidence that in the experiments at low α-catenin concentration we observe interactions with single α-β-catenin heterodimers. In fact, the 5.5 nm periodicity that we observe in our position data (Fig. 2d-f) can be observed only if one α-catenin molecule is interacting with actin, whereas α-catenin dimers or multiple molecules positioned randomly on the surface would average out this precise distribution. Therefore, it is very unlikely that single molecule experiments are affected by the presence of GST dimers.

At higher α-catenin concentration, we expect to have multiple α-catenin molecules interacting with actin. It remains possible that two adjacent α-catenin molecules might be bound to two β-catenin dimerized through the GST tag, which might possibly be arranged differently from two adjacent α-β-catenin heterodimers that are not dimerized through the GST tag. However, the present experimental arrangement does not allow us to control how α-β-catenin heterodimers are distributed on the coverslip surface and if and how particular arrangements of the molecules might affect their cooperative behavior. Understanding the details of this aspect is out of the scope of the present work and further studies would be needed to assess it.

Supplementary References

1. Heier, J. A., Dickinson, D. J. & Kwiatkowski, A. V. Measuring Protein Binding to F-actin by Co-sedimentation. *J. Vis. Exp.* e55613 (2017). doi:10.3791/55613
2. Rimm, D. L., Koslov, E. R., Kebriaei, P., Cianci, C. D. & Morrow, J. S. α1(E)-catenin is an actin-binding and -bundling protein mediating the attachment of F-actin to the membrane adhesion complex. *Proc. Natl. Acad. Sci. U. S. A.* **92**, 8813–8817 (1995).
3. Hansen, S. D. *et al.* αE-catenin actin-binding domain alters actin filament conformation and regulates binding of nucleation and disassembly factors. *Mol. Biol. Cell* **24**, 3710–20 (2013).
4. Lapetina, S. & Gil-Henn, H. A guide to simple, direct, and quantitative in vitro binding assays. *J. Biol. Methods* **4**, e62 (2017).
5. Pokutta, S., Choi, H.-J., Ahlsen, G., Hansen, S. D. & Weis, W. I. Structural and thermodynamic characterization of cadherin·β-catenin·α-catenin complex formation. *J. Biol. Chem.* **289**, 13589–601 (2014).
6. Howard, J. *Mechanics of motor proteins and the cytoskeleton*. (Sinauer Associates, Inc. Publisher, 2001).
7. Capitanio, M. *et al.* Ultrafast force-clamp spectroscopy of single molecules reveals load dependence of myosin working stroke. *Nat. Methods* **9**, 1013–1019 (2012).
8. Drees, F., Pokutta, S., Yamada, S., Nelson, W. J. & Weis, W. I. α-Catenin Is a Molecular Switch that Binds E-Cadherin-β-Catenin and Regulates Actin-Filament Assembly. *Cell* **123**, 903–915 (2005).
9. Rangarajan, E. S. & Izard, T. Dimer asymmetry defines α-catenin interactions. *Nat. Struct. Mol. Biol.* **20**, 188–193 (2013).
10. Koslov, E. R. *et al.* α -Catenin Can Form Asymmetric Homodimeric Complexes and / or Heterodimeric Complexes with β -Catenin. *J. Biol. Chem.* **272**, 27301–27306 (1997).
