# Supplementary material for: α-catenin switches between a slip and an asymmetric catch bond with F-actin to cooperatively regulate cell junction fluidity
Source: Nat Commun. 2022 Mar 3;13:1146. doi: 10.1038/s41467-022-28779-7 (PMC8894357; doi:10.1038/s41467-022-28779-7)
Supplement: Supplementary file 3 — Reporting Summary [file 41467_2022_28779_MOESM3_ESM.pdf]

nature research

Corresponding author(s): Marco Capitanio

Last updated by author(s): Sep 3, 2020

Reporting Summary

Nature Research wishes to improve the reproducibility of the work that we publish. This form provides structure for consistency and transparency in reporting. For further information on Nature Research policies, see our Editorial Policies and the Editorial Policy Checklist.

Statistics

For all statistical analyses, confirm that the following items are present in the figure legend, table legend, main text, or Methods section.

n/a	Confirmed	
[ ]	[x]	The exact sample size (*n*) for each experimental group/condition, given as a discrete number and unit of measurement
[ ]	[x]	A statement on whether measurements were taken from distinct samples or whether the same sample was measured repeatedly
[x]	[ ]	The statistical test(s) used AND whether they are one- or two-sided<br>*Only common tests should be described solely by name; describe more complex techniques in the Methods section.*
[x]	[ ]	A description of all covariates tested
[x]	[ ]	A description of any assumptions or corrections, such as tests of normality and adjustment for multiple comparisons
[ ]	[x]	A full description of the statistical parameters including central tendency (e.g. means) or other basic estimates (e.g. regression coefficient) AND variation (e.g. standard deviation) or associated estimates of uncertainty (e.g. confidence intervals)
[x]	[ ]	For null hypothesis testing, the test statistic (e.g. *F*, *t*, *r*) with confidence intervals, effect sizes, degrees of freedom and *P* value noted<br>*Give P values as exact values whenever suitable.*
[x]	[ ]	For Bayesian analysis, information on the choice of priors and Markov chain Monte Carlo settings
[x]	[ ]	For hierarchical and complex designs, identification of the appropriate level for tests and full reporting of outcomes
[x]	[ ]	Estimates of effect sizes (e.g. Cohen's *d*, Pearson's *r*), indicating how they were calculated

Our web collection on statistics for biologists contains articles on many of the points above.

Software and code

Policy information about availability of computer code

Data collection	Labview 8
Data analysis	Matlab R2016, Origin Pro 9, ImageJ 1.53

For manuscripts utilizing custom algorithms or software that are central to the research but not yet described in published literature, software must be made available to editors and reviewers. We strongly encourage code deposition in a community repository (e.g. GitHub). See the Nature Research guidelines for submitting code & software for further information.

Data

Policy information about availability of data

All manuscripts must include a data availability statement. This statement should provide the following information, where applicable:

- Accession codes, unique identifiers, or web links for publicly available datasets
- A list of figures that have associated raw data
- A description of any restrictions on data availability

The data generated in this study – duration and step size of single interactions that were used to generate lifetime plots (Fig. 1c, 2b, 3b, 4b-c, Supplementary Fig. 1a, 6), duty ratio plots (Fig. 5d-f), and step size distributions (Fig. 1g-j, 2c-d, 3c-f, Supplementary Fig. 3, 5, 8), as well as raw data of densitometry analysis in pull down experiments (Supplementary Fig. 11 and 12) – are provided in the Source Data file. The raw position and force data of trapping experiments are available upon request to the authors owing to the large size and proprietary format.

Field-specific reporting

Please select the one below that is the best fit for your research. If you are not sure, read the appropriate sections before making your selection.

[x] Life sciences [ ] Behavioural & social sciences [ ] Ecological, evolutionary & environmental sciences

For a reference copy of the document with all sections, see nature.com/documents/nr-reporting-summary-flat.pdf

Life sciences study design

All studies must disclose on these points even when the disclosure is negative.

Sample size	In single molecule optical trapping experiments, the sample size was 1 molecule. Sample size was determined as follows: approximately 1 in 4 silica beads showed interaction with the actin filament, providing evidence that the large majority of the beads contained at most a single catenin molecule.<br>In experiments with multiple alpha-beta catenin heterodimers, we estimated that between 5 and 10 molecules were interactin with a single actin filament, as discussed in the paper.<br>In experiments with oriented actin filaments, the lifetime data was derived from the average of 3 molecules for the single alpha-catenin homodimer and from the average of 3 ensembles of molecules for the multiple alpha-beta-catenin heterodimers.<br>The point of single molecule experiments is assure that data comes from 1 molecule at a time, and avoid averaging over multiple molecules, which would cancel out single-molecule behaviour. We checked the reproducibility of the results over several tens of molecules, and, when appropriate (like in the oriented dumbbell experiments) performed averages of lifetime data.
Data exclusions	No data was excluded
Replication	All attempts at veryfing reproducibility were successfull. Each experiment was replicated at least 10 times
Randomization	Randomization is not applicable to single molecule studies, it is used in clinical studies.
Blinding	Blinding is not applicable to single molecule studies, it is used in clinical studies.

Reporting for specific materials, systems and methods

We require information from authors about some types of materials, experimental systems and methods used in many studies. Here, indicate whether each material, system or method listed is relevant to your study. If you are not sure if a list item applies to your research, read the appropriate section before selecting a response.

Materials & experimental systems

n/a	Involved in the study	
[x]	[ ]	Antibodies
[x]	[ ]	Eukaryotic cell lines
[x]	[ ]	Palaeontology and archaeology
[x]	[ ]	Animals and other organisms
[x]	[ ]	Human research participants
[x]	[ ]	Clinical data
[x]	[ ]	Dual use research of concern

Methods

n/a	Involved in the study	
[x]	[ ]	ChIP-seq
[x]	[ ]	Flow cytometry
[x]	[ ]	MRI-based neuroimaging
